# Supplementary material for: Omnipresence of Partitiviruses in Rice Aggregate Sheath Spot Symptom-Associated Fungal Isolates from Paddies in Thailand
Source: Viruses. 2021 Nov 12;13(11):2269. doi: 10.3390/v13112269 (PMC8625198; doi:10.3390/v13112269)
Supplement: Supplementary file 1 [file viruses-13-02269-s001.zip › viruses-1445784-supplementary.pdf]

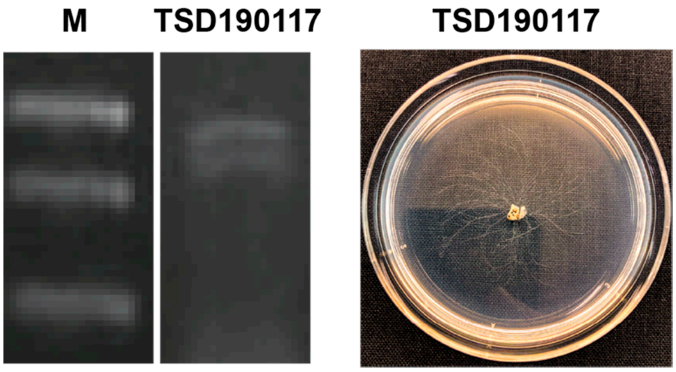

Figure S1. dsRNA profile and colony morphology of *Rhizoctonia solani* TSD190117. Strain TSD190117 exhibits severe growth reduction which might be associated with the presence of dsRNA with the estimated length of ≥2 kbp. The bands appeared on the left panel indicate molecular size marker of dsDNAs (2.5, 2.0 and 15 kbp, downward).

Table S1. List of primers used in this study.

Primer names	DNA sequences
ITS4	TCCTCCGCTTATTGATATGC
ITS5	GGAAGTAAAAGTCGTAACAAGG
dN6	CCTGAATTCGGATCCTCCNNNNNN
Amp	CCTGAATTCGGATCCTCC
RACE PV1T442-CP5'-R	CTGACGCAGATAAGCGTGCT
RACE PV1T442-CP3'-F	GCGTAACCTGGAACGTGCAT
RACE PV1T442-RdRp5'-R	GGGTTATCGCTAGTGTTTGGC
RACE PV1T442-RdRp3'-F	TTGATCAACGTCGGATCGTCG
RACE PV2T442-CP5'-R	CCTTCCTTGGTGAATTTGGC
RACE PV2T442-CP3'-F	GGTAAATGGCAGGGTCAGC
RACE PV2T442-RdRp3'-F	TGCGCTTCAGCGAATAGATC
RACE PV2T442-RdRp5'-R	GGAACTGGATGTGTGACTCC
RACE PV3T505-CP5'-R	GAATGGTTCTGCTGCTGCTG
RACE PV3T505-CP3'-F	GAGCGTAATTCTTGCCTTTAGC
RACE PV3T505-RdRp5'-R	GGTTCGCCTACGCTAGTTG
RACE PV3T505-RdRp3'-F	ATCAGGGAAGAGAACAGCGC
RACE PV4T123-RdRp5'-R	GTCGAAAGGTTCGACGTTGC
RACE PV4T123-RdRp3'-F	GGTATTCGGAAACTCACCTGAC
RACE PV4T123-CP5'-R	GTCGTCATCATCTTCTGGTCG
RACE PV4T123-CP3'-F	CCTTCTGGGAAGTCACCGAC
RACE PV5T123-RdRp5'-R	GTTTAGCTTGTGAGCCAGGC
RACE PV5T123-RdRp3'-F	CCAAGCTAGCATGGGATGCA
RACE PV5T123-CP5'-R	GGCCTGTCTAGCTTGTGCAG
RACE PV5T123-CP3'-F	GTTGGTGAAATCCCTGAATCTGG
RACE PV6T123-RdRp5'-R	GGTCACCCTTTCGTAAAGCTTC
RACE PV6T123-RdRp3'-F	GCTGCGGTATCGCATATGC
RACE PV6T123-CP5'-R	TCAGCGTCATCCTTTCCAGC
RACE PV6T123-CP3'-F	CCTCGTTCACTACCGATCGC

Table S2. BLASTn analysis of the fungal ITS fragments and the presence of dsRNAs.

Species	Isolate	Blastn		DsRNA presence
		Query (%)	Identity (%)	
Rhizoctonia oryzae-sativae				
	TSD 190101	99	99.41	
	TSD 190102	100	99.44	
	TSD 190103	100	99.58	√
	TSD 190106	100	99.85	√
	TSD 190107	99	99.86	
	TSD 190108	100	99.3	√
	TSD 190111	100	99.3	
	TSD 190113	100	99.44	√
	TSD 190118	99	98.88	
	TSD 190119	100	99.44	√
	TSD 190123	100	99.58	√
	TSD 190124	98	99.29	
	TSD 190125	98	99.86	√
	TSD 190129	100	99.44	
	TSD 190132	99	99.3	
	TSD 190133	98	99.3	√
	TSD 190136	97	98.45	√
	TSD 190137	98	99.43	
	TSD 190140	100	99.4	
	TSD 190142	96	99.86	
	TSD 190145	99	99.43	
	TSD 190202	100	99.55	
	TSM 190304	100	93.58	
	TSM 190305	99	99.71	
	TSM 190306	95	90.65	
	TSM 190307	86	84.05	
	TSM 190309	100	99.71	
	TSS 190401	100	86.81	√
	TSS 190436	99	99.3	
	TSS 190437	85	83.03	
	TSS 190440	86	84.5	
	TSS 190442	89	85.6	√
	TSS 190505	96	99.72	√
	TSS 190508	98	99.15	
	TSS 190538	97	98.59	
	TSS 190551	100	99.85	
	TSS 190554	100	99.44	
Rhizoctonia solani				
	TSD 190117	100	89.52	√
Rhizoctonia zeae				
	TSS 190513	89	85.6	√
Bipolaris sivanesaniana				
	TSD 190127	100	99.67	
	TSS 190406	99	99.67	
	TSS 190409	99	99.67	
	TSS 190413	99	99.51	
	TSS 190435	99	99.67	
	TSS 190439	99	99.5	

Species	Isolate	Blastn		DsRNA presence
		Query (%)	Identity (%)	
Gaeumannomyces oryzinus				
	TSD 190104	100	99.64	
	TSD 190105	99	99.13	
	TSD 190130	99	99.82	
	TSD 190138	100	99.63	
	TSD 190143	99	99.64	
Fusarium proliferatum				
	TSD 190120	100	99.81	
	TSS 190541	100	100	
	TSS 190545	100	100	
	TSS 190550	100	100	
Rhizoctonia oryzae				
	TSS 190517	99	93.92	√
	TSS 190523	100	99.23	
	TSS 190528	100	99.13	
Achroiostachys saccharicxola				
	TSS 190509	99	99.3	
	TSS 190514	99	99.65	
	TSS 190529	99	99.82	
Nigrospora oryzae				
	TSD 190144	99	98.88	√
	TSD 190210	99	99.82	
	TSS 190532	84	100	
Nigrospora sphaerica				
	TSD 190109	99	97.57	√
	TSD 190206	99	99.09	
Sclerotium hydrophilum				
	TSD 190209	78	84.9	
	TSD 190301	99	95.99	
Stachybotrys cf. elegans				
	TSS 190548	99	100	
	TSS 190542	99	99.06	
Setosphaeria rostrata				
	TSS 190415	99	99.35	
	TSS 190537	99	100	
Fusarium incarnatum				
	TSD 190114	99	99.82	
Fusarium equiseti				
	TSS 190526	100	100	
Fusarium sacchari				
	TSS 190539	99	100	
Bipolaris oryzae				
	TSS 190434	100	100	
Curvularia beasleyi				
	TSS 190515	100	99.65	
Simplicillium lamellicola				
	TSM 190303	99	99.68	
Sarocladium oryzae				
	TSS 190440	97	99..83	

Table S3. Full name and acronym of viruses used in phylogenetic analyses.

Acronym	Name	Accession number	
		RdRp	CP
HetPV1	Heterobasidion RNA virus 1	YP_009508049	YP_009508050
HetPV2	Heterobasidion partitivirus 2	YP_009508061	YP_009508062
RfPV	Rhizoctonia fumigata partitivirus	AJE25830	AJE25831
RsPV4	Rhizoctonia dsRNA virus 4	ATN23967	ATN23968
WCCV1	White clover cryptic virus 1	Q64FP0	Q64FN9
CCV	Carrot cryptic virus	YP_009508046	YP_009508045
BCV1	Beet cryptic virus 1	YP_002308574	YP_002308575
RnPV9	Rosellinia necatrix partitivirus 9	BBB86797	BBB86798
CpCV1	Chondrostereum purpureum cryptic virus 1	YP_009508236	YP_009508235
FvBv	Flammulina velutipes browning virus	YP_009508048	YP_009508047
MsAPV2	Medicago sativa alphapartitivirus 2	QBC36014	QBC36015
RosPV1	Rhizoctonia oryzae-sativae partitivirus 1	AYV61425	AYV61426
HetRV2	Heterobasidion partitivirus 2	YP_009508061	YP_009508062
AHV2H	Atkinsonella hypoxylon virus isolate 2H	Q85055	Q85056
RsPV6	Rhizoctonia solani partitivirus 6	QGA67322	QGA67323
SaPV1	Sodiomyces alkalinus partitivirus 1	ATQ64297	ATQ64296
RnPV8	Rosellinia necatrix partitivirus 8	BBC21044	BBC21045
PoV1	Pleurotus ostreatus virus 1	YP_227355	YP_227354
DCV2	Dill cryptic virus 2	YP_007891054	YP_007891055
CCCV2	Crimson clover cryptic virus 2	YP_009508059	YP_009508060
RCCV2	Red clover cryptic virus 2	YP_007889823	YP_007889824
HTCV2	Hop trefoil cryptic virus 2	YP_007889825	YP_007889826
FCV	Fig cryptic virus	YP_004429258	YP_004429259
BCV2	Beet cryptic virus 2	QCF59322	QCF59321
BCV3	Beet cryptic virus 3	YP_009665971	N/A
PCV1	Pepper cryptic virus 1	YP_009466859	YP_009466860
PCV2	Pepper cryptic virus 2	YP_009351838	YP_009351848
WcV2	Wuhan cricket virus 2	KX884243	KX884245
HplV11	Hubei partiti-like virus 11	YP_009329875	YP_009329874
BsPV3	Beauveria bassiana partitivirus 3	MN116721	MN116720
CePV1	Colletotrichum eremochloae partitivirus 1	AZT88590	AZT88591
PaP-LV1	Penicillium aurantiogriseum partiti-like virus	KT601103	KT601104

CSpV1	Cryptosporidium parvum virus 1	O15925	O15926
PsV-F	Penicillium stoloniferum virus F	YP_271922	YP_271923
FsV1	Fusarium solani virus 1	NP_624350	NP_624351
PsV-S	Penicillium stoloniferum virus S	YP_052856	YP_052857
AoV	Aspergillus ochraceous virus	YP_009665972	YP_009665973
GaRVMS1	Gremmeniella abietina RNA virus MS1	NP_659027	NP_659028
OPV1	Ophiostoma partitivirus 1	YP_009508238<br>NP_116716	YP_009508237
DdV1	Discula destructiva virus 1		NP_116742
BdPV1	Botryosphaeria dothidea partitivirus 1	AGZ84316	AGZ84317
FePV1	Fusarium equiseti partitivirus 1	MT659122	MT659123
AfPV2	Aspergillus flavus partitivirus 2	UAW09571	UAW09572
AtPV1	Alternaria alternata partitivirus 1	KY352402	KY352403
EfPV1	Epichloe festucae virus 1	YP_009508253	YP_009508252
MoV1	Magnaporthe oryzae virus 1	YP_122352	YP_122351

N/A Not available.

Table S4. dsRNA detection during the partitivirus-curing experiment.

**Stage**	**Method**	**Partitivirus detection (Pos/Total)**		**Total colonies** [e]
		5 mg/L cycloheximide	10 mg/L cycloheximide	
Screening [a]	1-step colony RT-PCR	4/24	3/23	47
Confirmation I [b]	dsRNA extraction	3/18	2/19	37
Confirmation II [c]	1-step colony RT-PCR	4/14	1/17	31
Final verification [d]	dsRNA extraction (more mycelium)	0/10	0/16	26

[a] Hyphal tipping was repeated five times, and the sixth hyphal tips were transferred to individual PDA plate, maintained for 5 d then subjected to RosPV1 and 2 detections.

[b] 200 mg of mycelium were used for dsRNA extraction for partitivirus infection.

[c] dsRNA negative variants were cultured on PDA for 10 d then subjected to another RosPV1 and 2 detections.

[d] 4 g of mycelium were used for dsRNA extraction for final confirmation of virus-cured variants.

[e] Total number of colonies tested in each stage.
